# Exposure of Intestinal Epithelial Cells to 2′-Fucosyllactose and CpG Enhances Galectin Release and Instructs Dendritic Cells to Drive Th1 and Regulatory-Type Immune Development

**DOI:** 10.3390/biom10050784

**Published:** 2020-05-19

**Authors:** Veronica Ayechu-Muruzabal, Saskia A. Overbeek, Atanaska I. Kostadinova, Bernd Stahl, Johan Garssen, Belinda van’t Land, Linette E.M. Willemsen

**Affiliations:** 1 Division of Pharmacology, Utrecht Institute for Pharmaceutical Sciences, Utrecht University, 3584 Utrecht, The Netherlands; v.ayechumuruzabal@uu.nl (V.A.-M.); Johan.Garssen@danone.com (J.G.); 2 Global Centre of Excellence in Immunology, Danone Nutricia Research B.V., 3584 CT Utrecht, The Netherlands; Saskia.OVERBEEK@nutricia.com (S.A.O.); atanaska.kostadinova@nutricia.com (A.I.K.); b.vantland@umcutrecht.nl (B.v.L.); 3 Global Centre of Excellence in Human Milk Research & Analytical Sciences, Danone Nutricia Research B.V., 3584 CT Utrecht, The Netherlands; bernd.stahl@danone.com; 4 Department of Chemical Biology & Drug Discovery, Utrecht Institute for Pharmaceutical Sciences, Utrecht University, 3584 CG Utrecht, The Netherlands; 5 Center for Translational Immunology, Wilhelmina Children’s Hospital, University Medical Center Utrecht, 3584 EA Utrecht, The Netherlands

**Keywords:** 1, 2′-fucosyllactose 2, non-digestible oligosaccharides 3, galectins 4, intestinal epithelial cells 5, dendritic cells 6, mucosal immunity

## Abstract

Intestinal epithelial cells (IEC) release immunomodulatory galectins upon exposure to CpG DNA (mimicking bacterial triggers) and short-chain galacto- and long-chain fructo-oligosaccharides (GF). This study aims to investigate the immunomodulatory properties of 2′-fucosyllactose (2′-FL), a non-digestible oligosaccharide (NDO) abundantly present in human milk, using a co-culture model developed to study the crosstalk between IEC and innate and adaptive immune cells. IECs, co-cultured with αCD3/CD28-activated peripheral blood mononuclear cells (PBMC), were apically exposed to NDOs and CpG, washed and co-cultured with immature monocyte-derived dendritic cells (moDC). Subsequently, moDC were co-cultured with naïve CD4+ T-cells. In the presence of CpG, both 2′-FL or GF-exposed IEC enhanced Th1-type IFNγ and regulatory IL-10 secretion of PBMCs, compared to CpG alone, while Th2-type IL-13 was reduced. Both NDOs increased IEC-derived galectin-3, -4, -9 and TGF-β1 of CpG-exposed IEC. Only galectin-9 correlated with all modified immune parameters and TGF-β1 secretion. MoDCs exposed to 2′-FL and CpG-conditioned IEC instructed IFNγ and IL-10 secretion by CD4+ T-cells, suggesting the development of a regulatory Th1 response. These results reveal that 2′-FL and GF could contribute to the mucosal immune development by supporting the effect of microbial CpG DNA associated with the modulation of epithelial galectin and TGF-β1 secretion.

## 1. Introduction

Non-digestible oligosaccharides (NDO) are abundantly present in human milk and consist of complex and diverse structures which vary during the course of lactation [1]. Over 160 different NDOs have been characterized so far, out of which 2′-fucosyllactose (2′-FL) is the most abundant in human milk of secretor-positive lactating women [2,3,4]. Up to 1% of NDO are absorbed and found in the systemic circulation [5] indicating that NDOs are able to interact with the immune cells present in circulation and thus, promote systemic effects. Infants fed formula supplemented with galacto-oligosaccharides and 2′-FL showed a lower inflammatory cytokine profile in serum, similar to the profile seen in breastfed infants [6]. Furthermore, 2′-FL was shown to support the maturation of intestinal epithelial cells (IEC) [7,8] as well as promoting immunomodulation through the interaction with immune cells [9,10,11,12].

The crosstalk between IEC, migratory dendritic cells (DC) and the resident immune cells is key to maintain the intestinal mucosal homeostasis and develop appropriate immune responses [13]. The migratory DC take up available antigens and travel to the mesenteric lymph nodes, where they can instruct naïve T-cells to develop into regulatory T-cells or effector T-cells which home back to the lamina propria via the bloodstream [14,15]. The function of DC can be modified by epithelial cell-derived mediators such as galectins or TGF-β [16,17,18]. Galectins are immunomodulatory glycan binding proteins highly expressed and secreted by epithelial cells [16,19] and thought to play a key role in infant immunity due to their ability to bind NDOs present in human milk [3]. Meanwhile, TGF-β is known for its contribution in sustaining immune homeostasis and mucosal protection [20,21], and can act in conjunction with galectins [22,23,24,25]. Epithelial release of these mediators may therefore affect both innate and adaptive mucosal immune functions. Hence, using dietary interventions to target IEC might be of interest to instruct immune development in the gastrointestinal tract.

Specific NDOs derived from milk or plant sources such as a 9:1 mixture of short-chain galacto- and long-chain fructo-oligosaccharides (GF) and *Bifidobacterium breve* M-16V were shown to reduce the development of allergic symptoms in mice by increasing galectin-9 levels locally, in the gastrointestinal tract, as well as systemically [26]. In addition, combined exposure to GF and synthetic CpG DNA or the CpG DNA derived from *Bifidobacterium breve* M-16V (TLR-9 agonists), resulted in increased IFNγ and IL-10 secretion in an IEC/peripheral blood mononuclear cell (PBMC) in vitro co-culture model [27,28]. These studies corroborate the ability of NDO in enhancing CpG induced immunomodulation as well as revealing the involvement of galectins in promoting such effects.

The aim of this study was to investigate the immunomodulatory effects elicited by 2′-FL and CpG-exposed IEC in a transwell IEC/PBMC co-culture model. Additionally, the crosstalk between IEC and monocyte-derived DC (moDC) was studied, followed by additional in vitro models to investigate the functional interaction of IEC-imprinted moDC with naïve CD4+ T-cells. Particularly, the association of epithelial-derived galectins and TGF-β1 secretion was analyzed regarding its contribution in the immune development.

## 2. Materials and Methods

### 2.1. Culture of Intestinal Epithelial Cells

Human colon adenocarcinoma HT-29 cell line (ATCC, HTB-38, Manassas, VA, USA) was used as IEC. The cells were cultured in 75 cm^2^ culture flasks (Greiner Bio-One, Alphen aan den Rijn, The Netherlands) using McCoy 5A medium (Gibco, Invitrogen, Carlsbad, CA, USA) supplemented with 10% heat inactivated fetal calf serum (FCS), penicillin (100 U/mL) and streptomycin (100 µg/mL) (Sigma-Aldrich, St. Louis, MO, USA). IEC were kept incubating at 37 °C and 5% CO_2_. The medium was refreshed every 2–3 days.

### 2.2. Peripheral Blood Mononuclear Cell Purification

Human PBMCs were isolated from buffy coats from healthy donors (Sanquin, Amsterdam, The Netherlands) by density gradient centrifugation (1000× *g*, 13 min), and washed with PBS (Lonza, Basel, Switzerland) supplemented with 2% FCS. Red blood cell lysis buffer was used to lyse the remaining erythrocytes (4.14 g NH_4_Cl, 0.5 g KHCO_3_, 18.6 mg Na_2_EDTA in 500 mL demi water, sterile filtered, pH = 7.4). The isolated PBMC were resuspended in RPMI 1640 supplemented with 2.5% FCS, penicillin (100 U/mL) and streptomycin (100 µg/mL).

### 2.3. Culture of Immature Monocyte-Derived Dendritic Cells

CD14^+^ cells were isolated from PBMC according to the manufacturer’s protocol by cell separation using a negative selection MACS kit (Miltenyi Biotec, Bergisch Gladbach, Germany). CD14^+^ cells were cultured for 7 days in RPMI 1640 medium supplemented with 10% FCS, penicillin (100 U/mL) and streptomycin (100 µg/mL) (Sigma-Aldrich), IL-4 (30–100 ng/mL) and GM-CSF (15–60 ng/mL) (both from Prospec, Rehovot, Israel). The medium was refreshed on days 2, 3 and 6 of culture. At day 7, immature moDC were collected.

### 2.4. IEC/PBMC and IEC/moDC Co-Culture Model Description

IEC were diluted 5 to 10 times based on surface area and seeded in 12-well transwell inserts (Costar Corning Incorporated, NY, USA) one week prior to the experiments. IEC were incubated at 37 °C, 5% CO_2_ and the medium was refreshed every 2–3 days. Confluent IEC monolayers were used to perform co-culture experiments.

#### 2.4.1. IEC/PBMC Model Description

**Figure 1:**
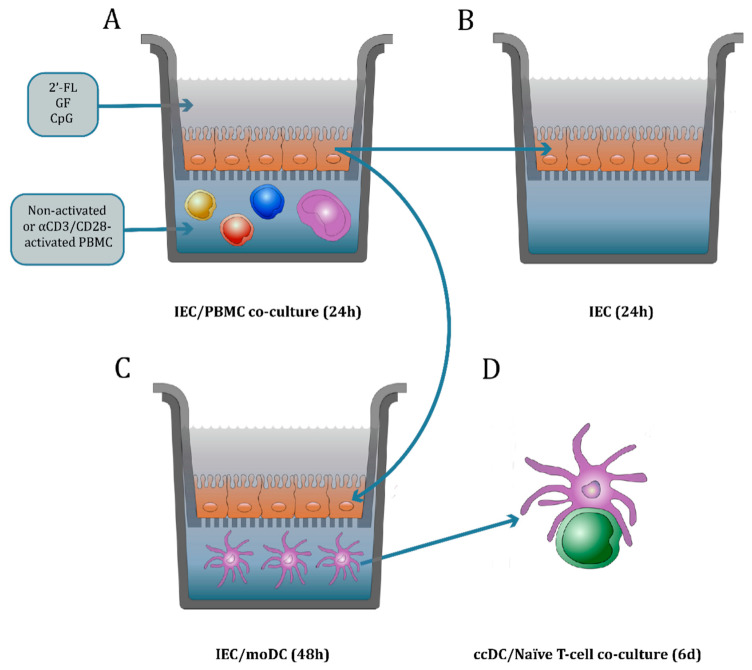
Co-culture model description. IEC were grown in 12-well transwell inserts until confluency and basolaterally exposed to either non-activated or αCD3/CD28-activated PBMC. Apically, IEC were conditioned with 2′-FL or GF in the presence or absence of CpG, a TLR9 agonist mimicking a bacterial trigger (**A**). After 24 h incubation, basolateral supernatant was collected to analyze the T-cell mediator release. The IEC were set apart and washed with PBS. Then, fresh medium was added and IEC were kept in incubation for an additional 24 h to study the IEC-derived mediator release (**B**). Alternatively, IEC were washed with PBS and co-cultured with immature moDC for 48 h (**C**). Then, the basolateral supernatant was collected where the mediator release was studied. Additionally, the phenotype of moDC after IEC/moDC co-culture was analyzed. Subsequently, conditioned moDC (ccDC) were exposed to naïve T-cells in an allogeneic DC/T-cell assay (**D**). After 5–6 days incubation, the cytokine release was measured in the supernatant.

IEC were basolaterally exposed to PBMC from healthy donors (2 × 10^6^ cells/mL) either activated with αCD3 and αCD28 (clone CLB-T3/2 and clone CLB-CD28 respectively, both 1:10.000, Sanquin,) or non-activated. 2′-FL or GF solutions (0.25−1% w/v; 2.5–10 mg/mL) either alone or in combination with CpG (0.5 µM CpG oligodeoxynucleotide M362 type C, Invivogen, San Diego, CA, USA) were added apically (Figure 1A). After 24 h IEC/PBMC incubation, basolateral supernatant was collected and stored at −20 °C for cytokine secretion analysis.

2′-FL produced by microbial fermentation with >90% purity may contain traces of glucose, fucose, lactose, 3′-FL, difucosyllactose, and water. GF is composed of a 9:1 mixture of short-chain galacto- and long-chain fructo-oligosaccharides. Galacto-oligosaccharides are obtained from lactose by enzymatic transglycosylation, while fructo-oligosaccharides are obtained from plant sources derived from inulin-type fructans. In order to evaluate the purity of 2′-FL and GF an endotoxin level assessment was performed by loading 25 µL of 1% NDO solution into an Endosafe^®^ cartridge, which was measured using an Endosafe^®^ Portable Test System (PTS) (Charles River Laboratories, Wilmington, MA, USA). Endotoxin levels from NDO were compared to a known LPS concentration (E. Coli O111:B4, Invivogen). The endotoxin levels from the NDO used in these studies were compared to a known LPS concentration. A concentration of 0.1 µg/mL LPS showed an equivalent of 0.76 EU/mL measured using the Endosafe^®^ test, while GF and 2′-FL gave 0.44 EU/mL and 0.88 EU/mL, respectively.

#### 2.4.2. IEC/moDC Co-Culture Model Description

Subsequent to IEC/PBMC co-culture, IEC cell monolayers were washed with PBS (Lonza,) and set apart in a new plate in the absence of PBMC for an additional 24 h (Figure 1B). After that, IEC-derived galectin-3, -4, -9 and TGF-β1 secretion was analyzed in the basolateral compartment. Alternatively, after IEC/PBMC co-culture the IEC cell monolayers were washed with PBS and co-cultured with immature moDC for 48 h in RPMI 1640 (Lonza) supplemented with 10% FCS, penicillin (100 U/mL) and streptomycin (100 µg/mL) (Figure 1C). After 48 h, the conditioned immature moDC (ccDC) were collected and their phenotype was studied. Additionally, the supernatant was collected and stored at −20 °C for cytokine secretion analysis.

### 2.5. DC/T-cell Co-Culture Model Description to Study the MoDC Function

CD4^+^CD45RA^+^ naïve T-cells were isolated from PBMC by negative selection using MACS separation kit, following the manufacturer’s protocol (Miltenyi Biotec), and resuspended in IMDM medium supplemented with 10% FCS, 20 µg/mL apotransferrine (Sigma-Aldrich), 50 µM β-mercaptoethanol (Sigma-Aldrich), penicillin (100 U/mL) and streptomycin (100 µg/mL). Naïve T-cells (1 × 10^6^) were co-cultured with ccDC (0.1 × 10^6^) from IEC/moDC culture, after IEC/PBMC exposure, in 24 well flat-bottom plates for 5 to 6 days in the presence of 1 ng/mL TGF-β (Prospec) (Figure 1D). After incubation, the supernatant was collected and stored at −20 °C for cytokine analysis. After the ccDC/T-cell co-culture, over 90% of the CD4+ T-cell were viable. The viability was not affected by exposure to NDO and/or CpG.

### 2.6. Enzyme-Linked Immunosorbent Assay (ELISA)

Supernatants from IEC/PBMC, IEC/moDC and ccDC/T-cell co-cultures were analyzed for cytokine and mediator secretion. Commercially available kits were used to determine IFNγ, IL-13, IL-17A (Thermo Fischer scientific, Waltham MA, USA), IL-10 (U-Cytech, Utrecht, The Netherlands), galectin-3, -4, -9 (R&D systems, Minneapolis, MN. USA) and IL-5 (Biolegend, San Diego, CA, USA) secretion according to the manufacturer’s protocol. Human galectin-4 or -9 were measured using antibody pairs (R&D systems). In short, high-binding Costar 9018 plates were incubated overnight at 4 °C with 0.75 µg/mL human galectin-4 or -9 affinity-purified polyclonal antibody. Non-specific binding was blocked with 1% BSA in PBS for 1 h, after which samples were incubated for 2 h at room temperature. After washing, biotinylated galectin-4 or -9 affinity-purified polyclonal antibodies (0.75 µg/mL) were added and incubated for 1 h. Then, plates were washed and streptavidin-HRP (R&D systems) was added and incubated for 1 h. After washing, tetramethylbenzidine was used as a substrate to develop the reaction (TMB, Thermo Fischer scientific), which was stopped with 1M H_2_SO_4_. Optical density was measured at 450 nm.

### 2.7. Flow Cytometry Analysis

After IEC/moDC co-culture, ccDC were collected and stained for flow cytometry analysis using CD11c-PerCP eFluor 710 (clone 3.9), CD14-APC (clone 61D3), HLA-DR-PE (clone LN3), CD80-FITC (clone 2D10.4) and CD86-PE Cyanine 7 (clone IT2.2) (all from eBioscience, San Diego, CA, USA). Viability was determined using Fixable Viability Dye 780-APC Cyanine 7 (eBioscience). Non-specific binding sites were blocked using PBS supplemented with 5% FCS before extracellular antibody staining. Flow cytometry measurements were done using BD FACS Canto II (Becton Dickinson, Franklin lakes, NJ, USA) and data were analyzed using Flowlogic software version 7 (Inivai Technologies, Mentone, VIC, Australia).

### 2.8. Statistical Analysis

Data were analyzed using Graphpad Prism 7 software (San Diego, CA, USA). Data were analyzed using one-way or two-way ANOVA followed by Bonferroni’s multiple comparison post hoc test on selected pairs. When data were not normally distributed, square root or logarithm transformation was applied prior to ANOVA analysis. In order to determine the strength of the association between specific mediators released, the Spearman’s rank correlation was applied. Data are represented as mean ± SEM of 6 to 12 independent PBMC donors. *p* values below 0.05 were considered of statistical significance.

## 3. Results

### 3.1. 2′-FL Enhances the Cytokine Release of Activated PBMC when Apically Exposed to IEC, in the Presence of CpG

**Figure 2:**
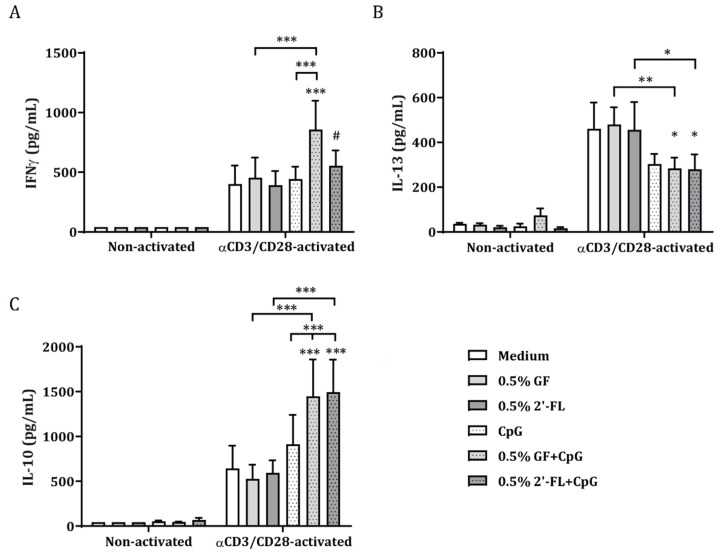
Cytokine secretion in IEC/PBMC co-culture after exposure to non-activated or αCD3/CD28-activated PBMC. IEC were basolaterally co-cultured with either αCD3/CD28-activated or non-activated PBMC for 24 h. Apically, IEC were exposed to 2′-FL or GF alone or in combination with CpG, a TLR9 agonist mimicking a bacterial trigger (Figure 1A). IFNγ (**A**), IL-13 (**B**) and IL-10 (**C**) concentrations were measured in the basolateral supernatant after IEC/PBMC co-culture. Data are represented as mean ± SEM of six independent PBMC donors. Two-way ANOVA and Bonferroni’s post-hoc tests were used to analyze statistical differences. Square root transformation was performed when data did not fit normal distribution (# *p* < 0.1, * *p* < 0.05, ** *p* < 0.01, *** *p* < 0.001).

To investigate the immunomodulatory effects of 2′-FL and GF in the presence or absence of CpG under homeostatic or inflammatory conditions, IEC were apically exposed to NDO and basolaterally co-cultured with non-activated or αCD3/CD28-activated PBMC for 24 h (Figure 1A). Culture of IEC with non-activated PBMC in the presence or absence of 2′-FL, GF and/or CpG did not promote the release of cytokines (Figure 2). Meanwhile, activation of PBMC with αCD3/CD28 resulted in increased IFNγ, IL-10 and IL-13 cytokine concentrations in the IEC/PBMC co-culture. These cytokines were not affected by exposure of IEC to 2′-FL, GF or CpG alone. However, upon apical exposure to 2′-FL or GF combined with CpG, IFNγ and IL-10 cytokine release was increased and IL-13 decreased, as compared to the medium control (Figure 2).

These results indicate that the immunomodulatory effects of the NDO described are exclusively elicited in the presence of an inflammatory milieu and upon availability of CpG, a TLR9 ligand representing bacterial CpG DNA. Hence, NDOs act synergistically with CpG to promote the immunomodulatory effects. Therefore, the following studies were performed using only αCD3/CD28-activated condition in IEC/PBMC co-culture.

### 3.2. Dose-Dependent Th1 and Regulatory-Type Immune Polarizing Effects of NDOs and CpG in the IEC/PBMC Co-Culture

Using the IEC/PBMC model (Figure 1A) we further studied whether 2′-FL has similar immunomodulatory properties as GF and the optimal dose at which these effects are elicited was established. Hence, dose-response studies were performed using CpG in combination with 0.25%, 0.5% and 1% NDO apically in the IEC/PBMC co-culture with αCD3/CD28-activated PBMC.

**Figure 3:**
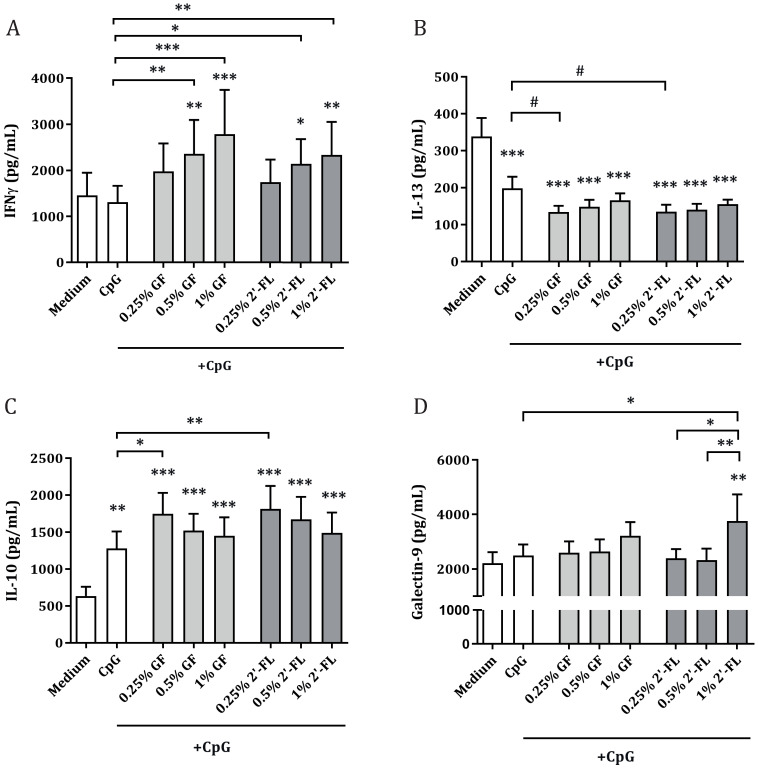
Cytokine and mediator secretion in IEC/PBMC co-culture. IEC were basolaterally exposed to αCD3/CD28-activated PBMC and apically to 0.25–1% NDO (2′-FL or GF) in combination with CpG (Figure 1A). After 24 h incubation, IFNγ (**A**), IL-13 (**B**), IL-10 (**C**) and galectin-9 (**D**) concentrations were measured in the basolateral supernatant. Data are represented as mean ± SEM of 7–8 independent PBMC donors (# *p* < 0.1, * *p* < 0.05, ** *p* < 0.01, *** *p* < 0.001).

Upon exposure of IEC to CpG alone, IL-13 concentrations were decreased, and IL-10 concentrations were increased as compared to medium control. IFNγ and galectin-9 concentrations were not affected (Figure 3). Combined exposure to 0.5% or higher concentrations of 2′-FL or GF with CpG resulted in significantly increased IFNγ concentrations compared to CpG alone, while the 0.25% dose did not show this effect. NDO, at a concentration of 0.25%, was however able to further increase IL-10 and tended to further reduce IL-13 concentrations (*p* = 0.08) compared to CpG alone, in IEC/PBMC co-culture (Figure 3). Only 1% 2′-FL, but not GF, combined with CpG increased galectin-9 concentrations in IEC/PBMC as compared to the medium. Exposure to 1% 2′-FL resulted in significantly increased galectin-9 concentrations compared to CpG alone and compared to the combination of CpG with lower 2′-FL concentrations. Galectin-9 release was positively correlated with IFNγ (r = 0.52, *p* < 0.0001) release and negatively with IL-10 (r = −0.38, *p* = 0.002) and IL-13 (r = −0.28, *p* = 0.036) secretion in IEC/PBMC co-culture (Figure S1). IL-5, a Th2 cytokine, showed similar results as IL-13, while no differences were found for IL-17A or TNFα concentrations (Figure S1).

### 3.3. Galectins and TGF-β1 Release by IEC Obtained from IEC/PBMC Co-Culture

To study the contribution of IEC to the immunomodulatory effects described in the IEC/PBMC model, conditioned epithelial cells derived from the co-culture model were washed and incubated with fresh medium for an additional 24 h (Figure 1B). Galectin-3, -4, -9 and TGF-β1 were measured in the basolateral compartment to study how 2′-FL and GF influence epithelial cell mediator release of CpG-exposed IEC in IEC/PBMC co-culture.

**Figure 4:**
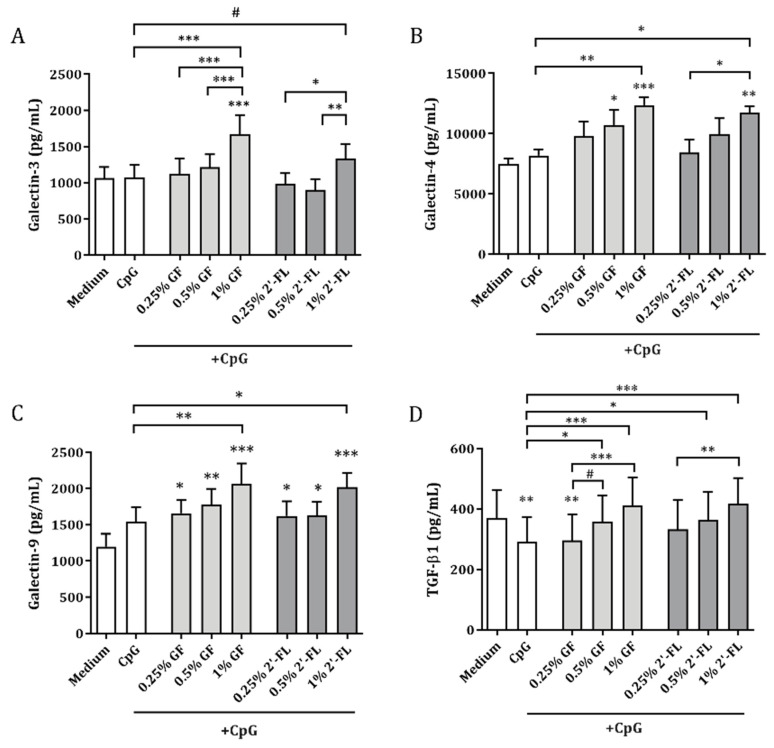
IEC-derived galectins and TGF-β1 secretion. IEC were washed after IEC/PBMC co-culture and incubated with fresh medium for an additional 24 h (Figure 1B). After the incubation period, IEC-derived galectin-3 (**A**), galectin-4 (**B**), galectin-9 (**C**) and TGF-β1 (**D**) were measured in the basolateral supernatant. Data are represented as mean ± SEM of 6–8 independent PBMC donors (# *p* < 0.1,* *p* < 0.05, ** *p* < 0.01, *** *p* < 0.001).

CpG did not affect IEC-derived galectin-3, -4 or -9 release but lowered TGF-β1 concentrations (Figure 4). In the presence of CpG, the highest dose of both NDOs (1% w/v) significantly increased galectin-3, -4 as well as -9 release from IEC, compared to CpG-exposed IEC (1% 2′-FL showed only a trend for galectin-3 release, *p* = 0.08). Exposure to 0.5% 2′-FL or GF in combination with CpG also resulted in a significant increase in TGF-β1 release compared to CpG alone. Galectin-3, -4 and/or TGF-β1 concentrations were increased after combined exposure to CpG and 1% 2′-FL or GF, as compared to 0.5% or 0.25% NDO (Figure 4). IEC-derived galectin-9, but not TGF-β1, correlated with galectin-3 (r = 0.4, *p* = 0.004) as well as with galectin-4 (r = 0.5, *p* = 0.0003) (Figure S2).

**Figure 5:**
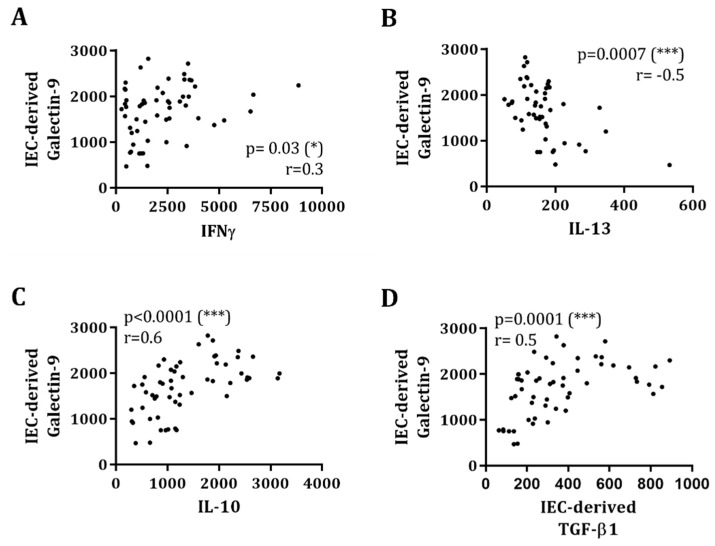
IEC-derived galectin-9 secretion correlates with IEC/PBMC co-culture cytokine release and IEC-derived TGF-β1. After 24 h IEC/PBMC co-culture, IFNγ, IL-13 and IL-10 concentrations were measured in the basolateral supernatant (Figure 1A and Figure 3). Thereafter, IEC were washed with PBS, the medium was refreshed and IEC incubated for additional 24 h (Figure 1B). After the incubation, the basolateral supernatant was collected and IEC-derived galectin-9 and TGF-β1 were measured (Figure 4). The correlation between IEC-derived galectin-9 and IFNγ (**A**), IL-13 (**B**), IL-10 (**C**) and TGF-β1 (**D**) release was tested using Spearman’s test (* *p* < 0.05, *** *p* < 0.001).

IEC-derived galectin-9 was positively correlated to IL-10 (r = 0.6, *p* < 0.0001), IFNγ (r = 0.3, *p* = 0.03) and IEC-derived TGF-β1 (r = 0.5, *p* < 0.0001), while being negatively correlated to IL-13 (r = −0.5, *p* = 0.0007), measured in the IEC/PBMC co-culture (Figure 5).

Beyond galectin-9, other epithelial-derived galectins were also found to correlate with the immune mediator production in the IEC/PBMC co-culture. Galectin-3 showed a strong positive correlation to IFNγ (r = 0.6, *p* < 0.0001) and a less strong negative correlation to IL-13 (r = −0.4, *p* = 0.01), while no correlation was found with IL-10 (r = 0.08, *p* = 0.6) (Figure S3). Meanwhile, galectin-4 concentrations were significantly correlated to IL-10 (r = 0.3, *p* = 0.02) but not to IFNγ (r = 0.1, *p* = 0.3) or IL-13 (r = −0.04, *p* = 0.8) (Figure S3).

### 3.4. Variations in the Expression of CD80 in MoDC after Co-Culture with Conditioned IEC

**Figure 6:**
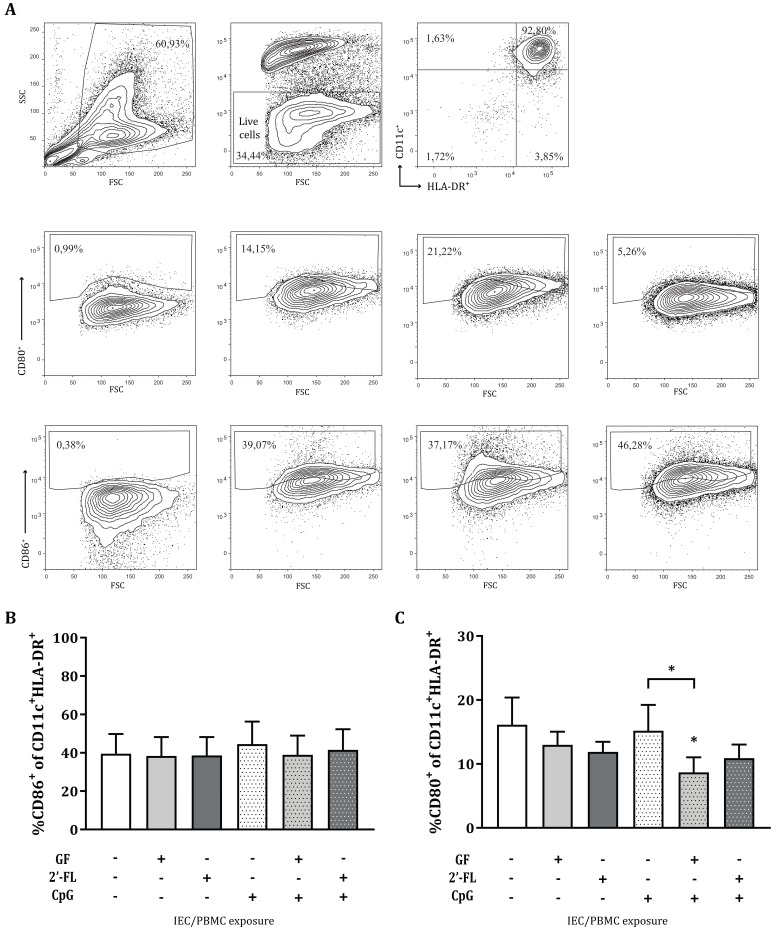
Phenotype of moDC after IEC/moDC co-culture. After exposure of IEC to 0.5% 2′-FL or GF, in the absence or presence of CpG, and co-culture with αCD3/CD28-activated PBMC, IEC were washed and co-cultured with immature moDC for 48 h (Figure 1C). The phenotype of moDC was studied after co-culture. Representative FACS plots are shown in (**A**). Expression of CD86^+^ (**B**) and CD80^+^ (**C**) was determined in the CD11c^+^HLA-DR^+^ population. Data are represented as mean ± SEM of eight independent moDC donors (* *p* < 0.05).

Subsequent to IEC/PBMC co-culture, the αCD3/CD28-activated PBMC were removed, and the conditioned IEC were washed and co-cultured with immature moDC in fresh medium for 48 h (Figure 1C). After this incubation, the phenotype of the ccDC was studied (Figure 6A). No significant differences were observed in the percentage of live cells, CD11c^+^HLA-DR^+^ cell populations or CD86^+^ expression by IEC-conditioned moDC from the IEC/moDC co-culture (Figure 6B). ccDC conditioned with IEC that were exposed to the combination of GF and CpG in the IEC/PBMC co-culture showed significantly decreased expression of CD80^+^ as compared to both CpG alone or medium control (Figure 6C).

Additionally, the release of galectin-3, -4, -9 and TGF-β1 was measured in the supernatant after IEC/moDC culture (Figure S4). No significant differences were observed in galectin-3 and TGF-β1 release. However, IEC-derived from IEC/PBMC co-cultures exposed to 0.5% GF alone or in combination with CpG resulted in a significant increase in galectin-4 release in the IEC/moDC co-culture. IEC from IEC/PBMC co-cultures exposed to CpG tended to increase galectin-9 concentrations in IEC/moDC co-culture which was not further affected by 2′-FL or GF (Figure S4).

### 3.5. CcDC Derived from IEC/moDC Co-Cultures after Conditioning of IEC with 2′FL and CpG in IEC/PBMC Co-Cultures Instruct IFNγ and IL-10 Production by Allogeneic CD4^+^ T-cells

**Figure 7:**
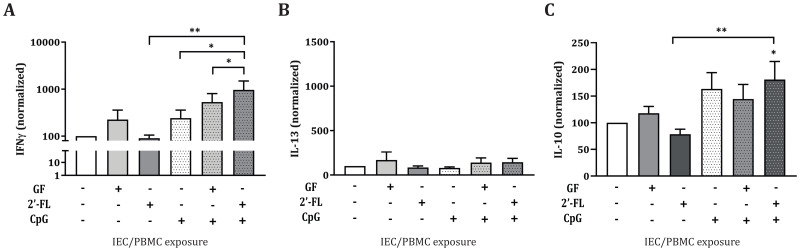
Cytokine secretion in ccDC/T-cell assay after moDC co-culture with conditioned IEC. Conditioned moDC (ccDC), previously exposed to conditioned IEC, were incubated with naïve T-cells for 5–6 days in an allogeneic ccDC/T-cell assay (Figure 1D). Afterwards, IFNγ (**A**), IL-13 (**B**) and IL-10 (**C**) were measured. Data were normalized per donor and represented as mean ± SEM from 5–12 independent PBMC donors (* *p* < 0.05, ** *p* < 0.01).

Conditioned moDC (ccDC) were then incubated with naïve T-cells for a maximum of 6 days in an allogeneic DC/T-cell assay (Figure 1D). IFNγ, IL-13 and IL-10 release was measured in the supernatant of the DC/T-cell culture (Figure 7). ccDC conditioned with IEC from IEC/PBMC co-cultures exposed to 2′-FL, GF or CpG alone did not show any effects in any of the cytokines measured. However, IEC from IEC/PBMC co-cultures exposed to 2′-FL in combination with CpG, showed increased IFNγ and IL-10 production in the ccDC/T-cell assay, compared to ccDC conditioned with IEC exposed to 2′-FL and/or CpG alone (Figure 7A,C). In addition, IFNγ production by T-cells was further increased when ccDC were conditioned to 2′-FL and CpG-exposed IEC from the IEC/PBMC as compared to GF and CpG-exposed conditions (Figure 7A). Meanwhile, IL-13 secretion was not affected (Figure 7B).

## 4. Discussion

NDOs in human milk are thought to modulate innate and adaptive immune properties and thereby promote the development of the mucosal immune system [29]. Direct immunomodulatory functions were previously described in vitro for 2′-FL through the interaction with IEC [7,8,10,12] as well as immune cells [6,7,11]. *In vivo*, a dietary intervention with 2′-FL resulted in an improved immune response to vaccination [9].

Previous studies have also described the mechanisms by which direct immunomodulatory effects of NDO such as GF could contribute to bacterial or synthetic CpG DNA in immune development in an in vitro IEC/PBMC co-culture model combining intestinal epithelial and immune cells [27,28,30,31,32]. These studies identified IEC-derived galectin-9 as an important factor contributing to immune development, which was confirmed in vivo by dietary intervention studies for food allergy prevention in mice [18,26]. In the present study, it was evaluated whether 2′-FL, one of the most abundant NDO present in human milk, could promote immunomodulatory effects under inflammatory conditions, using an in vitro IEC/PBMC co-culture model [27,28,30,31,32]. Moreover, the relation between IEC-derived galectin release and the immunomodulatory effects was addressed.

The current study shows that when PBMC are activated by means of αCD3/CD28, mimicking an inflammatory milieu, the secretion of immunomodulatory cytokines is boosted, as opposed to the non-activated condition (Figure 2). This was also seen in previous studies using the same model [30,32]. Apical exposure of IEC to 2′-FL or GF in combination with CpG was able to further increase Th1-type cytokine IFNγ as well as regulatory-type cytokine IL-10 secretion, while suppressing Th2-type cytokine IL-13 (Figure 3). This suggests that 2′-FL, as well as GF, can promote immunomodulatory effects in the IEC/PBMC model, which indicates that exposure to specific NDOs and CpG might contribute to the promotion of immune development. However, only exposure to high concentrations of 2′-FL resulted in increased galectin-9 concentrations already at 24 h after co-culture, as opposed to GF-exposed conditions, which did not show this effect (Figure 3). Following the dose-dependent studies in the IEC/PBMC co-culture experiments, the dose of 0.5% NDO was chosen for the subsequent studies, since this dose, when combined with CpG, increased Th1-type IFNγ and regulatory cytokine IL-10, while reducing the concentration of Th2-type IL-13.

2′-FL is composed of a fucose moiety linked to galactose and glucose while GF is a NDO mixture mainly composed of glucose bound to multiple galactose or multiple fructose residues, respectively. In addition, 2′-FL has a lower degree of polymerization as compared to GF. Previous studies showed that the cytokine profile can be affected by the type [33] as well as the chain length [34] of specific NDOs. Longer oligosaccharide chains might be able to interact with more than one receptor (or receptors located more distantly), forming a cluster, while shorter chains might interact only with receptors located around them. The differences in degree of polymerization, and thus chain length, of the studied NDOs, may be responsible for the distinct galectin-9 secretion found in the IEC/PBMC model.

Galectins are thought to play a key role in infant immunity due to their ability to bind NDOs present in human milk [3]. In particular, the ability of 2′-FL to bind galectin-9 and galectin-3 was previously described by Hirabayashi et al. using frontal affinity chromatography [35]. Galectins are soluble lectins that can also be secreted and consequently function as innate and adaptive immune modulators by binding several receptors on immune cells such as TIM-3 and CD44 in addition to binding specific glycosylation patterns on immune cells [16,36]. Galectins are secreted by several cell types, among which IEC are known to be a rich source [19]. In this study, exposure to both 2′-FL and GF, in combination with CpG, showed enhanced IEC-derived galectin-9 release (Figure 4). This was opposed to galectin-9 secretion 24 h after IEC/PBMC co-culture, which was not increased by exposure to GF and CpG, but only by 2′-FL and CpG (Figure 3). Although knowledge regarding the mechanism of action or receptors used by NDO in eliciting the direct immunomodulatory effects remains to be further developed, IEC-derived galectin-9 concentrations were correlated with IFNγ, IL-10 and IL-13 concentrations in the IEC/PBMC model (Figure 5). This indicates a role of galectin-9 in the immunomodulatory effects elicited by 2′-FL and GF. These results are in line with previous studies where the blocking of IEC-derived galectin-9 resulted in a reduction of IFNγ and IL-10 secretion and/or increase in IL-13 [27,28].

Beyond galectin-9, other galectins are also known to participate in diverse immune processes. Galectin-3 and -4 have shown anti-inflammatory properties by inhibiting mucosal inflammation in a colitis model [37,38]. The current study shows that IEC-derived galectin-3 and -4 were increased after exposure to 2′-FL or GF in combination with CpG (Figure 4). Galectin-3 concentrations were correlated to IFNγ and IL-13 but not to IL-10 secretion in the IEC/PBMC co-culture (Figure S3). Meanwhile, galectin-4 release was correlated only to IL-10 production.

The secretion of IEC-derived galectin-9 was correlated with IEC-derived galectin-3 and -4 (Figure S2) as well as with the cytokines secreted in the IEC/PBMC model (Figure 5), which might strengthen the idea that not only galectin-9, but also other galectins, might have contributed to the immunomodulatory effects seen in the IEC/PBMC model. Nevertheless, although the secretion of galectin-3 and -4 was increased after NDO and CpG exposure, epithelial-derived galectin-9 was the only galectin found to be correlated with the modulated IFNγ, IL-10, IL-13 secretion by PBMC as well as epithelial-derived TGF-β1, which reinforces the role of galectin-9 as a key factor in immunomodulation and thereby in immune development. The ability of galectin-9 in promoting immune regulation has also been described before, which substantiates the contribution of galectins in the regulation of immune homeostasis [23,24].

Furthermore, IEC-derived galectin-9 secretion was found to be strongly positively correlated with IEC-derived TGF-β1 (Figure 5). This supports the idea that both mediators act synergistically in the promotion of the differentiation of regulatory T-cells [22,23,24]. Conversely, TGF-β1 concentrations did not correlate to IEC-derived galectin-3 and -4 (Figure S2). In line with our results, the relation between epithelial galectin-9 and TGF-β1 mediator release, and the immunomodulatory effects elicited by combined exposure to GF and CpG in the IEC/PBMC model, has been previously described [25,27]. The formation of galectin-glycan lattices is known to influence cell signaling processes. All three galectins were found to be involved in cell signaling processes such as lipid raft stabilization and apical targeting of glycoproteins [39]. As a result of NDO and CpG exposure, IEC might have been able to increase signal transduction and thus promote mucosal immune homeostasis, maybe through the formation of galectin-glycan lattices. In agreement with the in vitro studies showing the involvement of epithelial-derived galectin-9 in promoting immunomodulation [25,27], *in vivo* NDO and *Bifidobacterium breve* were also shown to effectively induce galectin-9 concentrations, associated with mucosal immune regulation [26]. This shows the translational value of this type of co-culture models.

In addition to the immunomodulatory effects studied, we were interested in understanding whether the imprinted IEC, derived from the IEC/PBMC co-culture and exposed to medium only, had the ability to instruct DC to promote specific immune responses. Upon antigen exposure, IEC release several mediators that are able to activate migratory DC and their migration to the mesenteric lymph nodes, where they can promote adaptive immune responses by interacting with T-cells [14,15]. Due to the proximity of the migratory DC with the epithelial layer, their phenotype can also be affected upon epithelial mediator release, even in the absence of direct contact. As a result of the ability of 2′-FL and GF to modify epithelial mediator release in combination with CpG, the moDC phenotype was studied after conditioned-IEC/moDC co-culture. Subsequently, the functionality of the conditioned moDC was assessed by co-incubation with CD4+ T-cells in an allogeneic DC/T-cell model. Interestingly, this study shows that in the presence of CpG, exposure of IEC to GF, but not 2′-FL, resulted in a decreased CD80 marker expression of conditioned DC in the IEC/moDC model (Figure 6C), pointing towards a lower activation status. The decreased expression in CD80 should be considered with caution due to the high background signal present in the staining. Prospective studies should be done using an Fc block to control for this technical issue. Moreover, IEC imprinted by exposure to 2′-FL and CpG, in the IEC/PBMC model, instructed moDC to promote increased IFNγ and IL-10 production in the allogeneic DC/T-cell model, as compared to CpG alone (Figure 7). Meanwhile, GF-exposed IEC did not give rise to this effector Th-cell response. These results indicate the ability of 2′-FL and CpG-exposed IEC to instruct moDC to drive the development of naïve T-cells into Th1 and regulatory-type effector cells.

This study supports the idea that exposure to NDO in early life might promote immune development under conditions such as inflammation and emphasizes the ability of IEC to educate DC in strengthening mucosal immune function.

By developing the models described in this manuscript, it was aimed to highlight the relevance of the crosstalk between epithelial cells and immune cells in immunomodulation. The HT-29 intestinal epithelial cell line used in these studies, however, is a colon adenocarcinoma cell line. Although they are unable to completely mimic the structural and functional complexity of the in vivo situation, they serve as useful tools to study intestinal processes to some extent, which in future studies needs to be confirmed using primary human epithelial cell models. Great efforts have been made in order to develop primary epithelial cell 3D and 2D organoid models which provide regional-specific properties and more closely resemble the physiology of the gastrointestinal tract. Both pluripotent stem cell-derived organoids and ex vivo intestinal enteroid and colonoid cultures are powerful tools to study the heterogeneity and multicellular organization of intestinal epithelial cells in the gastrointestinal tract [40,41]. Beyond providing a physical barrier, IEC actively participate in diverse functions involving, among others, the immune system. The interactions between IEC and immune cells are key processes in immune homeostasis. The next step would therefore be to combine these primary epithelial cultures with immune cells as was already conducted with macrophages [42]. Beyond macrophages, the lamina propria consists of many other innate and adaptive immune cells which, preferably, could be isolated from intestinal tissues as well [43].

Despite its restrictions, the HT-29 transwell co-culture model was previously shown to have predictive value as the intervention with NDO not only identified an immunomodulatory role for galectin-9 in this in vitro model, but this was confirmed in murine models for food allergy. Furthermore, in infants with atopic dermatitis, formula milk containing NDO was capable of enhancing serum galectin-9 levels in association with symptom reduction [26], which supports the translational value of the HT-29 transwell co-culture model to the human situation. Our aim was to use the HT-29 transwell co-culture model as a first step of a sequence of models to illustrate the relevance of studying the interaction between structural cells and immune cells, thereby confirming the contribution of epithelial cells to modify innate and adaptive immune responses. The HT-29 transwell co-culture model could serve as a complementary model for future studies using primary 2D cultured enteroids.

## 5. Conclusions

This study reveals that both 2′-FL and GF can promote immunomodulatory effects under inflammatory conditions upon combined exposure with bacterial CpG DNA through the modulation of IEC function. These immunomodulatory effects were associated with the release of galectins and TGF-β1 by IEC. Thus, our study emphasizes the importance of understanding epithelial mediator release, such as galectins and TGF-β1, and their role in the mucosal immune development.

Additionally, we describe a possible role of 2′-FL and CpG-exposed IEC in instructing DC to drive naïve T-cell development. Future research should be directed towards further understanding of the mechanism of action by which these effects occur.

Prospective studies using 2D cultured human organoids will be needed to further validate the results discussed in this manuscript.

## Supplementary Materials

The following are available online at https://www.mdpi.com/2218-273X/10/5/784/s1, Figure S1: Cytokine release and correlations in IEC/PBMC co-culture, Figure S2: Correlations between IEC-derived mediator release, Figure S3: Correlations of IEC-derived mediator release and cytokine release in the IEC/PBMC co-culture, Figure S4: Cytokine concentrations in IEC/moDC co-culture.
