# Supplementary material for: Exposure of Intestinal Epithelial Cells to 2′-Fucosyllactose and CpG Enhances Galectin Release and Instructs Dendritic Cells to Drive Th1 and Regulatory-Type Immune Development
Source: Biomolecules. 2020 May 19;10(5):784. doi: 10.3390/biom10050784 (PMC7278199; doi:10.3390/biom10050784)
Supplement: Supplementary file 1 [file biomolecules-10-00784-s001.pdf]

Supplementary materials

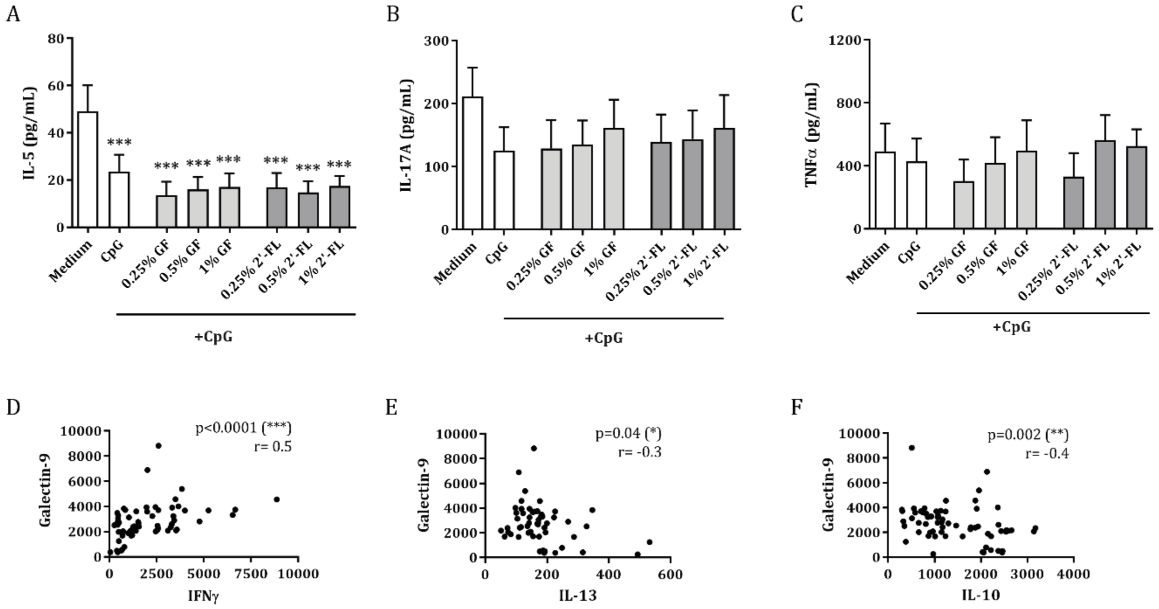

Figure S1. Cytokine release and correlations in IEC/PBMC co-culture. IEC were basolaterally exposed to αCD3/CD28-activated PBMC and apically to 0.25-1% NDO (2'-FL or GF) in combination with CpG (Figure 1A). After 24 hours incubation, IL-5 (**A**), IL-17A (**B**) and TNFα (**C**) concentrations were measured in the basolateral supernatant. Additionally, the correlations between galectin-9 and IFNγ (**D**), IL-13 (**E**) or IL-10 (**F**) concentrations were studied in the IEC/PBMC co-culture. Data are represented as mean ± SEM of six independent PBMC donors *(* $p < 0.05$, ** $p < 0.01$, *** $p < 0.001$).*

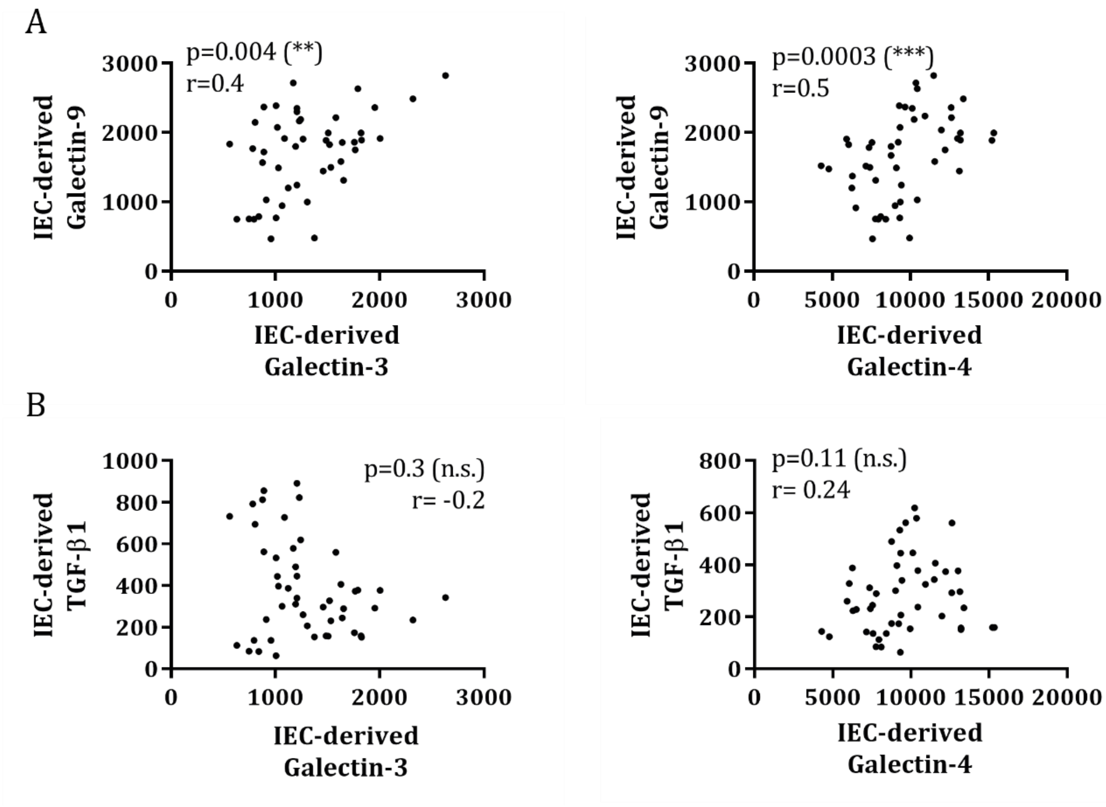

Figure S2. Correlations between IEC-derived mediator release. IEC were washed after IEC/PBMC co-culture. IEC-derived mediator release was studied by measuring galectin-3, -4, -9 and TGF-β1 concentrations in the basolateral supernatant (Figure 1B). Correlations between galectin-9 (**A**) and TGF-β1 (**B**) with galetin-3 and -4 are shown. Correlation was tested using Spearman's correlation coefficient *(** $p < 0.01$, *** $p < 0.001$)*.

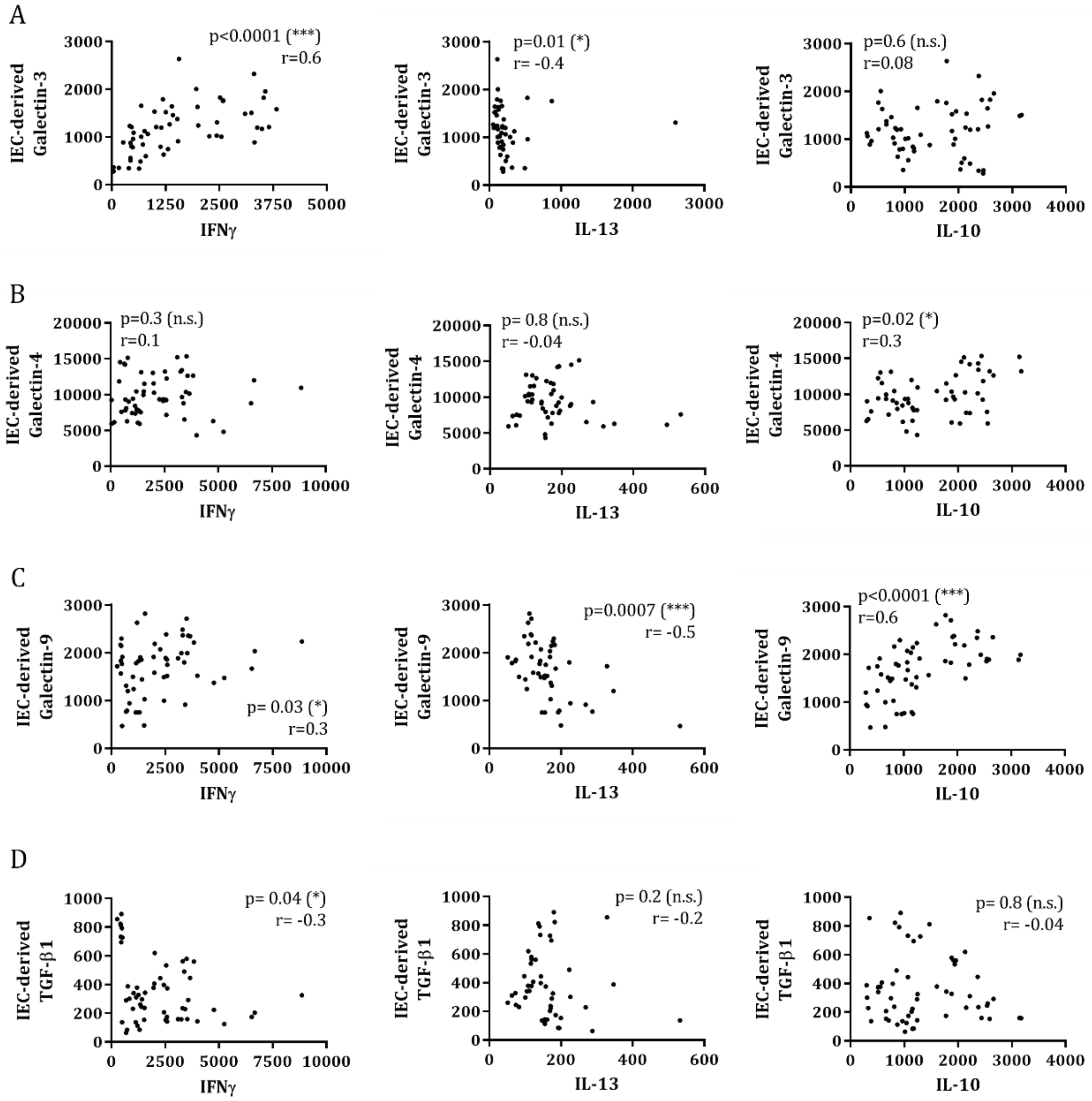

Figure S3. Correlations of IEC-derived mediator and cytokine release in the IEC/PBMC co-culture. IEC-derived mediator release galectin-3 (**A**), galectin-4 (**B**), galectin-9 (**C**) and TGF-β1 (**D**) were correlated to the concentrations of cytokines in IEC/PBMC (IFNγ, IL-13 and IL-10). Correlations were tested using Spearman's correlation coefficient *(* p < 0.05, *** p < 0.001).*

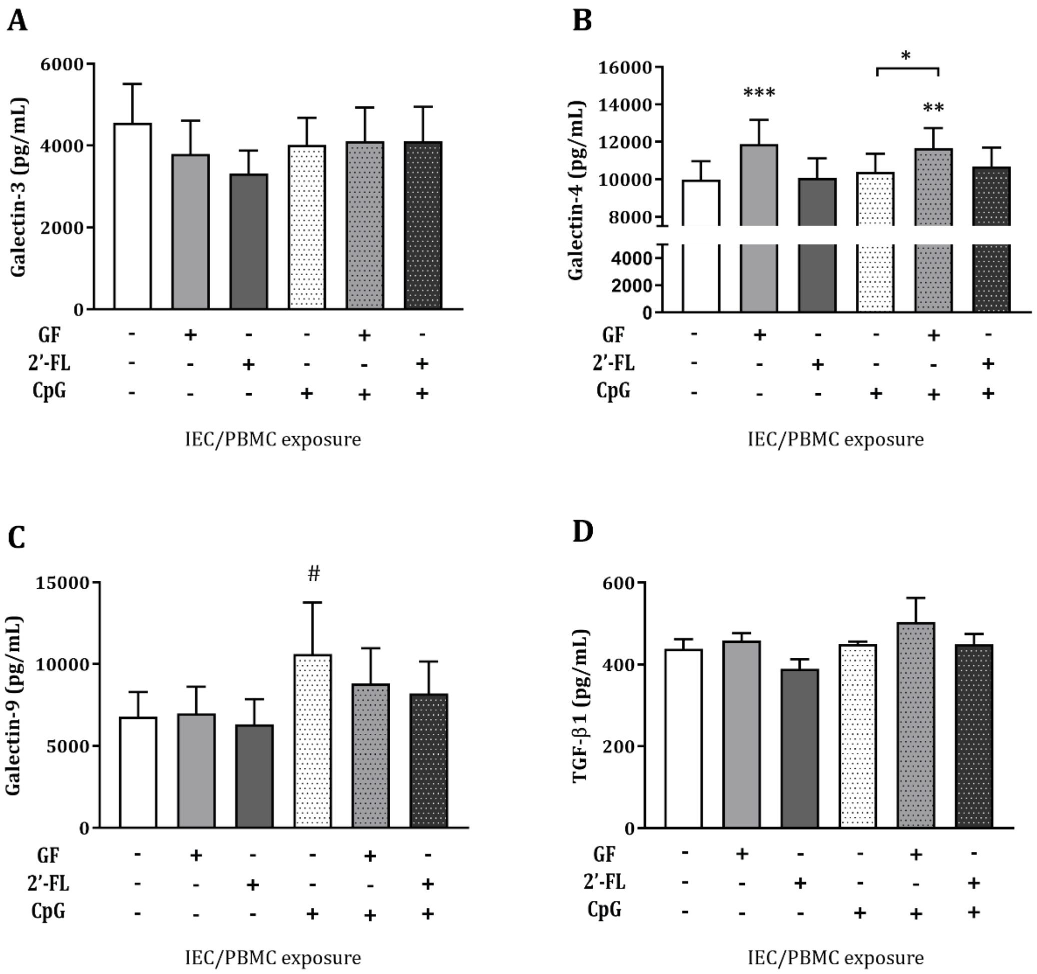

Figure S4. Cytokine concentrations in IEC/moDC co-culture. Conditioned IEC were co-cultured with immature moDC for 48 hours (Figure 1C). After incubation, concentrations of galectin-3 (**A**), galectin-4 (**B**), galectin-9 (**C**) and TGF-β1 (**D**) were measured in the basolateral supernatant. Results are represented as mean ± SEM from six independent PBMC donors *(# $p < 0.01$, * $p < 0.05$, ** $p < 0.01$, *** $p < 0.001$).*
